# Hepatocyte growth factor in cerebrospinal fluid differentiates community-acquired or nosocomial septic meningitis from other causes of pleocytosis

**DOI:** 10.1186/s12987-015-0020-z

**Published:** 2015-09-25

**Authors:** Amir Ramezani, Katarina Nägga, Oskar Hansson, Johanna Lönn, Johanna Sjöwall, Fateme Katoozian, Sepahdar Mansouri, Fariba Nayeri

**Affiliations:** Department of Neurosurgery, Region Östergötland, University Hospital, Linköping University, Linköping, Sweden; Clinical Memory Research Unit, Department of Clinical Sciences Malmö, Lund University, Malmö, Sweden; Department of Biomedicine, School of Health and Medical Sciences, Örebro University, Örebro, Sweden; Clinic of Infectious Diseases, Region Östergötland, University Hospital, Linköping University, 58185 Linköping, Sweden; The Institute of Protein Environment Affinity Surveys (PEAS Institut), Linköping, Sweden

**Keywords:** Septic meningitis, Aseptic, Alzheimer’s disease, HGF, Neuroborreliosis, Nosocomial infection

## Abstract

**Background:**

Due to anatomical restrictions, the inflammatory response to intracerebral bacterial infections exposes swollen brain tissues to pressure and ischemia, resulting in life-threatening damage. Rapid diagnosis and immediate empirical antibiotic therapy is highly important. However, diagnosing meningitis in patients after neurosurgery is complicated, due to brain tissue damage and changes in cerebrospinal fluid (CSF) caused by surgery. Hepatocyte growth factor (HGF) is a local, acute-phase protein with healing properties. Previous studies on community-acquired septic meningitis reported high levels of intrathecally produced HGF. The present study focused on nosocomial meningitis in assessing the levels of HGF in the CSF.

**Methods:**

HGF concentrations (ELISA) and HGF binding to receptors; c-Met receptor and heparan sulfate proteoglycan were determined in CSF samples (surface plasmon resonance). CSF samples from patients with community-acquired or nosocomial meningitis (217 samples from 135 patients) were compared to those from controls without signs of cerebral nervous system involvement (N = 36) and patients with Alzheimer’s disease (N = 20).

**Results:**

Compared to samples from patients that had undergone neurosurgery and had other infectious diseases, CSF samples from patients with nosocomial meningitis had significantly higher HGF concentrations (*p* < 0.001) and binding affinity to c-Met (*p* < 0.001) and HSPG (*p* = 0.043) receptors. The sensitivity and specificity to identify nosocomial septic meningitis were 69.7 and 93.4 %, respectively. The HGF concentration and binding affinity to HGF receptors were significantly higher in CSF from patients with community-acquired septic meningitis compared to patients with aseptic (viral and subacute) meningitis as well as controls (*p* < 0.001). The sensitivity and specificity to identify community-acquired septic meningitis were 95.4 and 95.7 %, respectively.

**Discussion:**

In febrile nosocomial infections that occurred post neurosurgery, HGF assessment could substantially improve the differentiation of meningitis from other infections and therefore might be a tool for rapid diagnosis, limiting injuries and guiding antibiotic therapy.

**Electronic supplementary material:**

The online version of this article (doi:10.1186/s12987-015-0020-z) contains supplementary material, which is available to authorized users.

## Background

An invasion of bacteria into the central nervous system (CNS) is followed by a rapidly evolving inflammatory process that affects the arachnoid space, the pia mater, and the cerebrospinal fluid (CSF). This condition leads to clinical symptoms of headache, fever, and meningism. The inflammatory response is caused by the release of various proinflammatory cytokines from meningeal cells into the subarachnoid space. As a result, neutrophils move into the subarachnoid space and cause pleocytosis in the CSF. The consequences include the breakdown of the blood–brain barrier, cerebral edema, reduced cerebral blood flow, focal areas of hypoperfusion, vascular thrombosis, ischemia, enhanced glucose metabolism via the anaerobic glycolytic pathway, and enhanced lactate accumulation in the brain and CSF [1]. Survival from this life-threatening condition depends on a rapid diagnosis and prompt empirical antibiotic therapy designed to cover the likely pathogens.

Other causes of febrile meningitis include acute viral meningitis and non-pyogenic meningitis, where the clinical picture is typically sub-acute or chronic. The diagnostic procedures consist of a lumbar puncture to analyze the CSF for cells and bacteria, microbiological cultures of blood and CSF, serological tests involving PCR and antigen-detection, and radiographic techniques [2]. In community-acquired meningitis, a combination of discriminating values from the CSF analysis can differentiate acute bacterial meningitis from other, non-ambulatory causes with quite high sensitivity [3].

Nosocomial meningitis can occur when brain surgical procedures are complicated by infection [2]. Due to underlying CNS disease processes and CNS devices in situ, the principal agents of nosocomial bacterial meningitis differ from the agents of community-acquired meningitis. For example, in nosocomial infections, slow growing, opportunistic microorganisms predominate. The CSF leukocyte profile is affected by intracerebral hemorrhage, and CSF lactate might be elevated, due to ischemia. Moreover, altered consciousness can make it difficult to establish a diagnosis in patients on ventilators that develop fever after neurosurgical operations. In other words, it is often difficult to determine whether the injured brain has been invaded by environmental bacterial flora.

Hepatocyte growth factor (HGF) is produced by mesenchymal cells during organ injury. It is produced as a single-chain precursor protein, and it is activated at the site of injury by proteolytic cleavage, resulting in a double-chained active form of HGF [4]. Active HGF stimulates cell division [4] and cell motility [5], and it promotes normal morphogenic structure [6] in epithelial cells adjacent to injured areas. Thus, HGF induces regeneration and repair of damaged tissue [7]. High levels of systemic HGF have been detected during injuries caused by infection [8]. In bacterial meningitis, pneumonia and acute bacterial gasteroenteritis, there is local production of HGF at the site of infection [9–11]. HGF produced locally during bacterial infection is biologically active [12, 13]. Application of biologically active HGF promoted healing of chronic leg ulcers in a pilot study [14]. Effective antibiotic therapy reduces systemic HGF levels during infection [15–17]. HGF might be regarded as a local acute phase protein with healing properties [18].

The quality of endogenous HGF binding to receptors can be assessed with surface plasmon resonance (SPR), an optical technique appropriate for clinical studies that can determine the affinity of a protein for several ligands [19]. SPR-based assessment of HGF binding affinity for its receptors, c-Met and heparan sulfate proteoglycan (HSPG), could rapidly and sensitively distinguish HGF variants with different biological activities [20–22].

A high level of HGF in the CSF has been reported in patients with Alzheimer’s disease, a chronic disorder characterized by progressive neuronal degeneration and deposits of amyloid plaques and neurofibrillary tangles [23]. Cerebral inflammation is also involved in the pathogenesis of Alzheimer’s disease [24, 25].

We have previously studied the concentration of HGF in CSF for patients with community-acquired meningitis [9]. We have also shown that the presence of biologically active HGF at the site of injury indicates an acute local inflammation [18]. The aim of the present study was to assess whether HGF concentrations and HGF binding affinity for its receptors might serve as markers to distinguish between meningitis associated with neurosurgery and other causes of infection. Therefore, we assessed HGF in patients with either community-acquired meningitis or neurosurgery-associated meningitis, and compared the results to either patients with Alzheimer’s disease or controls with normal CSF.

## Methods

### CSF samples

**Fig. 1:**
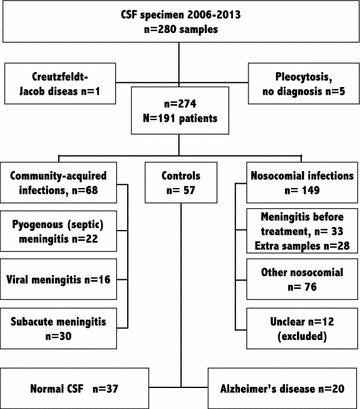
Flow chart of the selection of CSF specimens from patient and control groups. Extra samples are the samples taken during stay on ward from patients with nosocomial meningitis

This study was performed in Linköping, Sweden. The CSF samples from patients with septic (pyogenic) and aseptic meningitis were collected at the University Hospital in Linköping, Sweden, over the 8-year period of 2006–2013. The CSF specimens from patients with community-acquired infection or controls were collected after lumbar punctures and handled promptly. In one patient the sample was collected from ventricular drain. The samples from patients that had undergone neurosurgical intervention were collected from ventricular drains. The samples were collected and centrifuged (3000×*g* for 15 min) and then stored in in polypropylene tubes at −70 °C until use. CSF samples from patients diagnosed with Alzheimer’s disease were obtained from the Memory Clinic, Skåne University Hospital in Malmö, Sweden. The Regional Ethics Committees in Linköping and Lund, respectively, approved the study protocols. Five patients with pleocytosis of unknown origin and one patient with Creutzfeldt-Jakob disease (positive western blotting for protein 14-3-3 in CSF) were excluded. The remaining samples (n = 274) were assigned to one of six groups, depending on the type of meningitis (Group 1–4) or controls (Group 5, 6) (Fig. 1). Please note the difference between the number of samples (n) and the number of patients (N) in each group (Additional file 1).

### CSF sample groups

#### Community-acquired meningitis

*Group 1: septic meningitis (n* *=* *22 samples)* Patients with community-acquired acute bacterial meningitis (N = 20) had a median age of 62 years (range 24–75; with 7 females). In 2 cases, samples were collected on two occasions (CSF samples, n = 22). The cases presented with fever, CSF polynuclear pleocytosis, and elevated CSF lactate and protein levels. The causes were identified as *Streptococcus pneumonia* (N = 6), *Haemophilus influenzae* (N = 2), *Listeria monocytogenes* (N = 1), *Streptococcus intermedius* (N = 3), *α*-*hemolytic streptococci* (N = 1), *rickettsial meningitis* (N = 1), *Coagulase*-*negative staphylococcus* (N = 1), *Cryptococcus neoformans* (N = 1), *Aspergillus* (N = 1), *Staphylococcus aureus* (N = 1), and unknown (N = 2), due to negative results in microbiological assessments.

*Group 2: Aseptic meningitis (n* *=* *46 samples)* Patients with viral or subacute meningitis or encephalitis were considered one group in statistical calculations. The laboratory findings included CSF pleocytosis, normal CSF lactate levels, and negative blood and CSF cultures.

- 15 patients had viral meningitis (median age 41 years, range 21–88, 7 females). Laboratory assessments revealed Herpes zoster (positive PCR; N = 4), enterovirus (positive PCR; N = 5), and negative results in the laboratory assessments (N = 6). In 1 case, samples were collected on two occasions (n = 16 samples).
- 30 samples were collected from patients with subacute meningitis or encephalitis (N = 30). Laboratory assessments revealed *Borrelia burgdorferi* (positive intrathecal anti-borrelia antibodies; N = 28), syphilis (positive antibodies in serum; N = 1), and highly suspected tuberculosis (The patient had been treated for tuberculosis, had clinical CNS symptoms, and pleocytosis but direct microscopy of CSF, PCR, and cultures were negative; was treated successfully with anti-tuberculosis medicine, N = 1).

### Nosocomial meningitis

CSF samples (n = 149) were collected from patients (N = 69) that were admitted to the Department of Neurosurgery, University Hospital in Linköping, Sweden. The patients had undergone a neurosurgical procedure, and the management was complicated with infection. Thus, several CSF specimens were collected from some patients on different occasions. Ventilator associated pneumonia and meningitis were the differential diagnoses of infection in the majority of cases. Antibiotic therapy was initiated or changed to broad spectrum antibiotics by the physician in charge, directly after cultures of blood, CSF, catheters, and drains were taken.

Because no golden standard is available to define the diagnosis in post neurosurgery patients that develop fever, among the CSF samples, two major groups were identified based on medical history, the clinical judgment of physician in charge, the follow-up and the diagnose code in medical records.

*Group 3: Nosocomial meningitis (n* *=* *33 samples)* CSF specimens (n = 61) were collected from patients (N = 29, median age, 62 years, range 21–76; 14 females) that had undergone brain tumor surgery (N = 8), intracerebral hemorrhage (N = 15), shunt dysfunction (N = 3), skull fracture (N = 2), or two different periods of disease (N = 1). In these patients, microbiological assessments yielded bacterial growth or CSF pleocytosis, and they were treated for bacterial meningitis. Prior to antibiotic therapy, 33 CSF samples were collected (in three cases, samples were collected on 2 occasions). The CSF cultures showed growth of *Coagulase*-*negative staphylococcus* (N = 7), *Propionibacterium acnes* (N = 5), *Enterococcus faecalis* (N = 2), *α*-*hemolytic streptococci* (N = 1), and *Candida glabrata* (N = 1). In blood cultures, we found *Pseudomonas aeroginosa* (N = 1) and *Lactococcus lactis* (N = 1). 12 patients had negative cultures.

In 13 patients, CSF samples were collected before and after therapy at 2–5 occasions (totally 42/61 specimens). Assessments of the 13 patient reports indicated two sub-groups:

- CSF samples were collected both before and after successful therapy (N = 7);
- CSF samples were collected from patients that failed therapy or from patients before therapy was initiated (N = 6).

*Group 4: Other nosocomial infections (n* *=* *76 samples)* CSF specimens (n = 76) were collected from patients (N = 37) (median age 55 years, range 0.5–81) that underwent neurosurgery for tumors (N = 6), cerebral hemorrhage (N = 23), shunt dysfunction (N = 5), fractures (N = 2), or surgical complications (N = 1). The course of treatment was complicated with bronchitis or pneumonia (N = 19, n = 50), other infections (N = 5, n = 7, urinary tract infection, skin and soft tissue infection) or unverified or treated infections (CSF samples obtained after 1 week treatment) (N = 13, n = 19).

Among the nosocomial infections, 12/149 CSF specimens were unclear cases (died on ward or were transferred to another hospital before a diagnosis was set) and were excluded from statistical calculations.

### Controls

*Group 5: Alzheimer’s disease (n* *=* *20)* CSF samples were collected from patients (N = 20) with a median age of 78.5 years (range 61–86, 15 females) that were diagnosed with Alzheimer’s disease at the Memory Clinic, Skåne University Hospital in Malmö. The patients had all undergone a routine lumbar puncture, cognitive testing, and computed tomography or magnetic resonance imaging of the brain as part of the dementia investigation. The revised DSM-III and NINDS-ADRDA criteria were used for diagnosis of Alzheimer’s dementia [26, 27].

*Group 6: Normal CSF (n* *=* *37)* This group consists of two subgroups, but it is considered one group in statistical calculations. One subgroup included patients (N = 19) with a median age of 50 years (range 24–74; 7 females, in one case, samples were collected on two occasions) (n = 20). These patients underwent lumbar puncture to rule out meningitis, and they had normal CSF. The other subgroup comprised patients (N = 17) with a median age of 58 years (range 48–78 years, 10 females). These patients had very subtle cognitive symptoms, and they were judged to be cognitively healthy after a dementia evaluation at the Memory Clinic, Skåne University Hospital in Malmö.

### Analysis of ligand-binding affinity with surface plasmon resonance

The biological activity of HGF was analyzed with SPR. We measured the binding affinity to HSPG (Sigma-Aldrich, St. Louis, MO, USA) and to a c-Met recombinant chimera (R&D Systems), as previously described [19]. Briefly, SPR measurements and ligand immobilization procedures were conducted with a 760-nm light beam emitted in a fully automatic, Biacore 2000 system (GE-Healthcare GmbH, Uppsala, Sweden), equipped with four flow cells. In all experiments, the flow cell temperature was 25 °C. The running buffer contained 0.01 M HEPES, pH 7.4, 0.15 M NaCl, 3 mM EDTA, and 0.005 % surfactant P20 (HBS-EP; GE-Healthcare GmbH). Ligands were coupled to carboxymethylated dextran CM5 chips (GE Healthcare GmbH) with conventional carbodiimide chemistry: 200 mM N-ethyl-N0-(3 diethylaminopropyl) carbodiimide and 50 mM N-hydroxysuccinimide. The activation time was 7 min, followed by a 7-min ligand injection. Deactivation of the remaining active esters was performed with a 7-min injection of ethanolamine/hydrochloride, pH 8.5. A flow rate of 5 μL/min was used in the immobilization and measurement procedures. The HSPG (Sigma-Aldrich, St. Louis, MO, USA) solution contained ≥400 μg/mL protein and 400 μg/mL glycosaminoglycan; this solution and the recombinant c-Met receptor chimera (100 µg/mL, R&D Systems, Minneapolis, MN, USA) were diluted 1:5 in 10 mM acetate buffer, pH 4.5. This buffer maintained the reaction below the isoelectric points of the proteins to enhance electrostatic interactions between the dextran matrix and the ligands. The contact time was 7 min, which resulted in immobilization levels between 200 and 1000 response units (RU).

CSF samples were thawed, centrifuged at 3000×*g* for 10 min, and diluted 1:1 in phosphate buffered saline solution (PBS, pH 7.4; Apoteket AB, Umeå, Sweden). CSF regeneration was induced by adding an equal volume of 1 M NaCl and 10 mM glycine, pH 2, followed by one injection of borate, pH 8.5. The positive control consisted of HGF produced by leukocytes from a healthy volunteer; the negative control was PBS. Both were included at the beginning and end of each run to confirm the reliability of the surfaces.

### Measurements of HGF concentrations in CSF

A specific enzyme-linked immunosorbent assay (ELISA) kit (Quantikine Human HGF immunoassay, minimum detectable limit: 0.04 ng/mL; R&D Systems, Minneapolis, MN, USA) was used to determine HGF concentrations in CSF, according to the manufacturer`s instructions. All measurements were performed with an ELISA reader (Expert96; AsysHitech GmbH, Eugendorf, Austria) at 450 nm, which was calibrated with the recombinant human HGF reference samples and the standards provided in the kit.

### Routine laboratory assessments

CSF was analyzed to determine parameter values at the Departments of Clinical Chemistry, University Hospitals, in Linköping and Malmö, Sweden. All cultures, PCR assays, antigen detection assays, and serological assessments were performed at the Department of Microbiology, University Hospital, Linköping, Sweden.

*CSF*-*cells* Performed by manual phase contrast microscopy (Zeiss) using Jessen Chamber for counting the number of erythrocytes and leukocytes (polymorphonuclear neutrophils and monocytes).

*CSF lactate* Performed using benchtop blood gas analyzer ABL 800 (Radiometer Medical ApS Denmark).

*Antigen detection* Antigen (*Streptococcus pneumonia*, *Haemophilus influenza*, *Nisseria meningitides* group A, group B**/***Escherichia coli* K1, group C and group Y/135, *Streptococcus agalactiae*) was detected by latex agglutination method Pastorex Meningitis™ (Bio-Rad, France).

*CSF culture* Performed in aerobe and anaerobe flasks as well as in Hematin plates.

*Lyme neuroborreliosis* Intrathecal antibodies against *Borrelia burgdorferi* were detected by capture Elisa technique.

*Virus detection PCR* Herpes virus type 1 and 2 (HSV1, HSV 2) and HZV DNA were detected by PCR. Human enterovirus (HEV) was analyzed by automated instrument (Cepheid GeneXpert).

### Statistical analysis

Because the HGF concentrations and binding affinity data were not normally distributed, we compared values before and after therapy with the Kruskal–Wallis test followed by the Mann–Whitney U test or the Wilcoxon matched pairs test. All analyses were performed with Graph Pad Prism version 5 (San Diego, CA, USA). Values are expressed as the median. P-values <0.05 were considered statistically significant. The sensitivity, specificity, positive predictive values, and negative predictive values were calculated manually. The intra- and inter-assay coefficient of variation (CV) for determination of HGF concentration was calculated manually and was found to be 12.9 and 11 %, respectively.

## Results

### CSF specimens

The community-acquired septic meningitis showed significantly higher HGF concentration (*p* = 0.0133), as well as HGF binding affinity to the c-Met and HSPG receptors (*p* = 0.0007 and *p* = 0.0009, respectively) compared to nosocomial meningitis. CSF samples from patients with septic meningitis (including both community-acquired and nosocomial) was significantly higher in HGF concentrations (*p* = 0.0014), HGF binding to HSPG (*p* < 0.0001), and HGF binding to c-Met (*p* < 0.0001) compared to samples from patients with aseptic (viral and subacute) meningitis. CSF samples from patients with septic meningitis was higher from samples from the control group (patients with normal CSF) and from patients with Alzheimer’s disease in HGF concentration (*p* < 0.0001, *p* = 0.0010, respectively), HGF binding to HSPG (*p* < 0.0001 and *p* = 0.9, respectively), and HGF binding to c-Met (both *p* < 0.0001).

Compared to samples from patients that had undergone neurosurgery and had other infectious diseases, CSF samples from patients with nosocomial meningitis had significantly higher HGF concentrations (*p* < 0.0001) and HGF binding affinity to c-Met (*p* < 0.0001) and HSPG (*p* = 0.043) receptors.

**Fig. 2:**
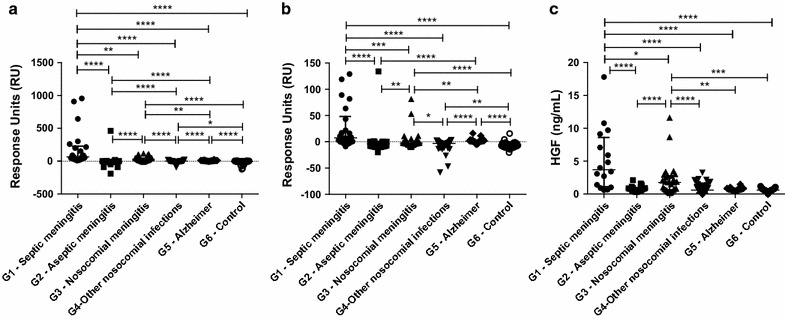
Properties of HGF derived from different CSF samples were analyzed by using surface plasmon and ELISA techniques. **a** Binding to c-Met receptors; **b** Binding to HSPG receptors; **c** HGF concentrations (median). * *p* < 0.05, ** *p* < 0.01, *** *p* < 0.001, **** *p* < 0.0001

CSF samples from patients with Alzheimer’s disease had significantly higher binding affinity to HSPG and c-Met compared to samples from patients with aseptic meningitis (*p* < 0.0001), samples from controls (*p* < 0.0001), and samples from patients that had undergone neurosurgery and had infections other than meningitis (*p* < 0.0001) (Table 1; Fig. 2).

**Table 1:** The CSF samples in groups (G1–G6)

Groups 1–6	Red blood cells (×10^9^)	White blood cells (×10^9^)	C-Met binding (RU)^a^	HSPG binding (RU)^a^	HGF (ng/ml)
Septic community-acquired meningitis (n = 22)	200 (0.1–49,000)	400 (3.6–8400)	56 (9.6–965.3)	6.7 (0–129)	3.08 (0.58–17.51)
Aseptic/Subacute meningitis (n = 47)	2.2 (0–1820)	58 (0.6–1042)	0 (0–462)	0 (0-134)	0.59 (0.18–2.1)
Septic nosocomial meningitis (n = 33)	7000 (1–75,000)	164 (0-2000)	27.3 (0–116.4)	0 (0–81.2)	2.04 (0.125–11.59)
Nosocomial other infections (n = 76)	3500 (0–350,000)	18 (0.2-545)	0 (0–25)	0 (0–3.8)	0.57 (0–3.26)
Alzheimer’s disease (n = 20)	nd^b^	nd^b^	7.4 (0–26.1)	1.3 (0–16.2)	0.71 (0.43–1.47)
Normal CSF (n = 37)	–	–	0 (0–7.1)	0 (0–15.7)	0.59 (0.02–1.16)

All values represent median (range)

^a^Negative values in surface plasmon resonance are reported as 0

^b^ *nd* not

### Estimations

We measured the binding of CSF-derived HGF to c-Met receptors in the SPR system. We used a cut off value of 10 response units (RU) to diagnose pyogenic meningitis in nosocomial (sensitivity 69.7 %, specificity 93.4 %, positive predictive value 82.1 % and negative predictive value 87.6 %) (Table 2a) and community-acquired infections (sensitivity 95.4 %, specificity 95.7 %, positive predictive value 91.3 %, negative predictive value 97.8 % (Table 2b).

**Table 2:** The sensitivity and specificity for the diagnostic accuracy of CSF-derived HGF binding affinity to c-Met receptor; a cut off value = 10.0 response units (RU) was used to diagnose septic meningitis in community-acquired and nosocomial meningitis

Sub-groups	Nosocomial fever		
C-met affinity	Meningitis	Other infections	Total
≥10	23	5	28
<10	10	71	81
Total	33	76	109

Sub-groups	Community acquired		
C-met affinity	Septic meningitis	Aseptic meningitis	Total
≥10	21	2	23
<10	1	45	46
Total	22	47	69

Values represent the numbers of patients in each group. The sensitivity and specificity of the diagnostic test were 69.7 and 93.4 %, respectively (a: nosocomial meningitis/other febrile infections post neurosurgery) and 95.4 % respective 95.7 % (b: community acquired septic/aseptic meningitis)

### Correlation analysis in nosocomial cases

Statistical dependence between the HGF variables (c-Met, HSPG, and ELISA) and white blood cells was tested in cases having nosocomial meningitis (G3) and other nosocomial infections (G4) by using Spearman’s rho. In these cases, the white blood cells count correlated to HGF amounts ELISA (0.44; p < 0.001), c-Met binding (0.56; p < 0.001), and to HSPG binding (0.28; p = 0.004). These results suggest that some diagnostic information is shared between white blood cell count and HGF variables, and, to elucidate the independent value of HGF, we further subdivided the 109 nosocomial cases into two groups according to WBC (<10 … and >10….). All 30 cases with a low WBC count had a low c-Met binding (<10 RU) and in this group 2 had septic meningitis (2/30 = 7 %). Fifty-one nosocomial cases had a high WBC count but a low c-Met binding and 8 of these had meningitis (8/51 = 16 %). Among the 28 cases with both a high WBC count and a high c-Met binding, 23 were diagnosed with septic meningitis (23/28 = 82 %). The difference in the proportion of cases with meningitis between the two last groups was statistically significant (p < 0.001) indicating that there is independent diagnostic information in c-Met binding (additional to that of WBC count).

Correlation between the HGF variables (c-Met, HSPG, and Elisa) and red blood cells (>500 × 10^9^, n = 132) was tested by using Spearman’s rho. In these cases, the correlations to HGF concentration Elisa, c-Met binding and HSPG binding were R = 0.12, 0.20 and 0.40 respectively (Table 3).

### Monitoring

**Fig. 3:**
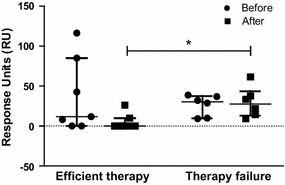
HGF binding to c-Met receptor in CSF was measured before (*circle*) and after (*square*) treatment in 13 cases with nosocomial meningitis. These were divided into groups based on whether they responded effectively to therapy (Efficient therapy, N = 7) or did not respond to therapy (Therapy failure, N = 6); * *p* < 0.05

In some patients with nosocomial meningitis (N = 13), CSF samples had been collected before and after antibiotic therapy. We found that CSF-derived HGF binding to c-Met receptors decreased after treatment in CSF samples from patients that responded to antibiotic treatment. However, the change did not achieve significance (N = 7, *p* = 0.06). In contrast, HGF binding to c-Met increased in CSF samples from patients that did not respond to therapy; again, the change was not statistically significant (N = 6, *p* = 0.07). However, the post-treatment levels of binding differed significantly between responder and non-responder sub-groups (*p* = 0.0153, Mann–Whitney test; Fig. 3).

## Discussion

In the present study, we show that an assessment of CSF-derived HGF binding to c-Met receptors can be used to substantially improve the differentiation between infection in the injured brain after surgery and other nosocomial infections, such as ventilator-associated pneumonia. In addition, this assessment can be used to differentiate between community-acquired septic meningitis and aseptic meningitis with high sensitivity and specificity.

Due to the challenges in establishing a diagnosis, and the lack of gold standards, physicians are motivated to treat suspected infections in serious diseases with broad-spectrum antibiotics. The emerging problems of multiple-resistance bacteria, high costs, and complications related to new antibiotics have called for diagnostic tests that can minimize antibiotic consumption.

Currently, the diagnosis of bacterial meningitis remains based on standard methods of direct microscopy, differential analyses of white blood cells, lactate, and protein, and cultures of blood and CSF [3]. However, post-neurosurgical infections are difficult to distinguish from the effects of neurosurgical procedures [28, 29]. For example, when patients develop fever within days after surgery, a determination of cells and lactate in the CSF cannot provide reliable information. Moreover, due to prophylaxis treatments, the cultures are negative in a large group of patients, and the presence of skin flora, like *Coagulase negative Staphylocococcus* or *Propionbacterium acnes*, may indicate either infection or contamination.

In the present study, we measured HGF concentration and receptor-binding affinity in CSF samples. Consistent with previous observations regarding presence and quality of HGF in body fluids during infection [30, 31], we found a significant difference in CSF-derived HGF concentrations between samples from patients with community-acquired septic meningitis and samples from those with aseptic meningitis. Furthermore, we observed that binding of CSF-derived HGF to the c-Met receptor could differentiate meningitis from other causes of fever in patients that had undergone neurosurgery; this assay had quite high specificity (=93 %), and it could rule out nosocomial meningitis in 88 % of cases (negative predictive value, 88 %; Table 2a). There was no statistically significant difference in the CSF-derived HGF concentrations before and after therapy but the levels were lower in cases that responded to antibiotics compared to levels in nonresponders (Fig. 3); therefore, in part, the pathological difference between damage caused by surgery and that caused by infection might be the presence or absence of external pathogens.

Previous studies have reported elevated concentrations of CSF-derived HGF in Alzheimer’s disease [32]. Recently, we reported high HGF concentrations, but low biological HGF activity during chronic inflammation [33]. In the present study, we observed that HGF binding affinity for HSPG, an indicator of HGF biological activity [21], was significantly lower in viral and subacute meningitis than in septic meningitis. Because it has been shown that inflammation is present in Alzheimer’s disease [25], we expected high HGF concentrations (ELISA), but low binding affinity to HGF receptors (HSPG, C-Met, SPR). Surprisingly, we observed that the HGF profile was similar to that in acute inflammation; in the CSF from patients with Alzheimer’s disease, the HGF binding affinity to HSPG was significantly higher than in the samples from all other groups, except for the samples from patients with community-acquired septic meningitis (Table 1). Proteoglycans possess diverse physiological roles, particularly in neural development, and are also implicated in the pathogenesis of neurodegenerative diseases [34–36]. Our observation of high binding affinity of CSF to HSPG in SPR might indicate the role of HSPG in etiology and pathophysiology of Alzheimer’s disease, not relating to presence or biological activity of HGF in CSF.

Limitations: The present work has limitations that should be addressed in future prospective and independent multicenter studies. The source of collection of CSF differed in community-acquired meningitis (lumbar puncture) from nosocomial infections after surgery (ventricular drains). As far as we know there are no reports about differences in concentration of circulating proteins in CSF collected by lumbar puncture or from ventricular drains. The major message of this study is the higher level of HGF in CSF in patients who suffered from nosocomial septic meningitis compared to other nosocomial infections and the method of collection of CSF did not differ between these patient groups.

The patients that had undergone neurosurgery had high red blood cell count in CSF that might indicate a passive transfer of HGF from blood into CSF [37]. There were no matched blood samples to calculate the blood–brain barrier damage and to assess the intrathecal production of HGF. However, in the previous study [9] we have reported intrathecal production of HGF during septic meningitis. The results show a significantly higher CSF HGF levels in nosocomial septic meningitis compared to other nosocomial infections in the post- neurosurgery patients that had low correlation to the number of cells in CSF.

**Table 3:** As shown the correlation between c-Met binding (cut-off 10 RU) and number of white blood cells in CSF (cut-off 10 x 10^9^) differs based on the number of cells

	White blood cells (×10^9^)		Total
	<10	≥10	
c-Met binding (RU)			
<10	30	51	81
≥10	0	28	28
Total	30	79	109

The samples were collected during several years and kept frozen until they were analyzed. We have previously studied the stability of HGF in blood and feces and observed that HGF was stable in these body fluids during longer storage and several freeze–thaw cycles [38, 39].

## Conclusions

Consistent with our previous studies, from the present study, we concluded that a determination of HGF in CSF might be used as an indicator, complementary to clinical status and routine laboratory findings, for diagnosing bacterial invasion into the CSF at an early stage of disease. The benefit of this kind of diagnosis is that it can limit the brain damage and guide the selection of an appropriate antibiotic therapy, particularly in nosocomial infections, where standard diagnostic methods are of limited value.

## Additional file

**Additional file 1.** In the Supplemental Material Section an Excel file with detailed information about the cases included in the study is presented.
